# Psychological Factors and Treatment Experiences Associated With Chemotherapy Side‐Effect Expectations and Symptom Severity: A Prospective Longitudinal Study

**DOI:** 10.1002/cam4.72117

**Published:** 2026-07-14

**Authors:** Miriam Grapp, Kristin Lichtenberg, Marcel Wilhelm, Hans‐Christoph Friederich, Till Johannes Bugaj

**Affiliations:** ^1^ Department of General Internal and Psychosomatic Medicine University Hospital Heidelberg Heidelberg Germany; ^2^ National Center for Tumour Diseases (NCT) University Hospital Heidelberg Heidelberg Germany; ^3^ Department of Clinical Psychology and Psychotherapy Philipps Universität Marburg Germany; ^4^ DZPG (German Centre for Mental Health–Partner Site Heidelberg/Mannheim/Ulm) Ulm Germany

**Keywords:** anxiety, cancer, chemotherapy, depression, fatigue, hope, oncology, self efficacy, treatment outcome

## Abstract

**Background:**

Patients' expectations may influence how chemotherapy side effects are perceived and could contribute to symptom amplification. Psychological distress and prior treatment experiences are assumed to shape these expectations, yet their interplay and impact on side‐effect burden remain unclear.

**Aims:**

This study examined how prior treatment experiences, psychological distress, hope, and self‐efficacy affect expectations of side effects and predict the severity of chemotherapy‐related symptoms such as nausea, peripheral neuropathy, and fatigue.

**Methods:**

In a prospective longitudinal study, 101 adult patients with cancer initiating outpatient chemotherapy were assessed at T1 (start of the second cycle) and T2 (8–12 weeks later). Expectations of side effects (G‐EEE), chemotherapy‐related symptom severity (GASE), hope (HHI‐D), self‐efficacy (CBI‐B‐D), and psychological distress (PHQ‐2, GAD‐2) were assessed using standardized questionnaires. Prior treatment experiences, sociodemographic and illness‐related variables were documented. Multiple linear regressions identified predictors of expectations and symptom severity.

**Results:**

Of 101 patients, 73 completed both assessments. Higher side effect expectations were predicted by prior chemotherapy, anxiety, lower hope, and negative treatment reports from others. At T2, depressive symptoms predicted neuropathy and fatigue; lower self‐efficacy predicted neuropathy; and lower hope predicted fatigue. Expectations improved model fit but did not independently predict symptom severity.

**Conclusions:**

Findings highlight the relevance of personal and vicarious treatment experiences, psychological distress, and resilience factors in shaping both expectations and patients' symptom experiences related to chemotherapy side effects. While expectations were linked to psychological variables, hope, self‐efficacy, and depression were stronger predictors of symptom severity. These patient characteristics may help identify patients who could benefit from supportive care and patient‐centered communication in oncology.

**Trial Registration:**

German Clinical Trials Register (DRKS00028791)

## 1 Background

Despite advances in personalized cancer therapies, chemotherapy remains a cornerstone of oncological treatment, playing a critical role in modern combination therapy [1]. Recent progress has focused on improving the tolerability of chemotherapy to reduce adverse effects and enhance patients' quality of life [2]. Nevertheless, side effects remain a significant clinical concern, negatively impacting both clinical outcomes and patients' quality of life [3]. The most commonly reported chemotherapy‐related side effects include fatigue, gastrointestinal symptoms, hair loss, and peripheral neuropathy [4].

Despite their common biological basis, patients receiving similar chemotherapy regimens often report substantial differences in the occurrence and severity of treatment‐related symptoms [5]. This variability suggests that patients' experiences of chemotherapy‐related symptoms may be influenced not only by treatment‐related factors but also by individual psychological and contextual characteristics.

These observations are reflected in symptom science frameworks such as the Theory of Unpleasant Symptoms (TOUS) [6], which conceptualizes symptoms as multidimensional subjective experiences shaped by interacting physiological, psychological, and situational factors [7, 8]. Within this framework, psychological variables such as anxiety, depression, hope, and self‐efficacy, as well as prior treatment experiences and social influences, may shape patients' perceptions and experiences of chemotherapy‐related side effects. The framework further emphasizes that symptoms are not isolated phenomena but dynamic experiences that may interact and influence patients' functioning and quality of life.

One psychological factor that has received increasing attention in this context is patients' expectations regarding treatment‐related side effects. Research on psychological processes related to patients' treatment expectations suggests that symptom experiences may be influenced not only by physiological treatment effects but also by how patients anticipate and interpret these effects [9]. Within this line of research, nocebo‐related mechanisms have been proposed as one explanation for how negative expectations may affect the perception, severity, and reporting of treatment‐related symptoms. Nocebo‐related mechanisms are commonly understood as adverse effects of negative expectations and contextual treatment factors [10]. In clinical settings, these mechanisms may not necessarily generate entirely new symptoms but may influence the perception, severity, and reporting of symptoms that are also attributable to the disease or its treatment [11]. A key driver is negative expectations, which can increase attentional focus on adverse outcomes [12].

Such expectations may arise from various sources. Personal experiences with similar treatments can lead to learned responses that reinforce symptom expectations. Communication with healthcare providers also plays a critical role, as negatively framed information or emphasis on side effects can amplify nocebo responses [13, 14]. Social and media influences further contribute: reports from peers or relatives, or observing others describe side effects can shape attitudes toward treatment and increase symptom reporting [15]. These expectation processes are supported by learning and neurobiological mechanisms. Experimental research shows that symptom expectations can be changed through associative learning, whereby contextual cues linked to prior treatment experiences may later influence symptom perception and symptom reporting [16]. Expectations are further shaped by prior information, context, and communication, and are dynamically updated through experience [17]. These processes are underpinned by neurobiological pathways involving central nervous system networks that modulate symptom perception and physiological responses [18].

Psychological factors such as anxiety, catastrophizing, and depression have also been consistently linked to heightened nocebo responses across medical contexts [12, 19]. From a cognitive perspective, depression and anxiety may influence expectation formation by altering how expectations are updated in response to new experiences. Expectations are shaped by prior beliefs and revised when actual experiences differ from what was expected. In depression, this updating process is biased: negative expectations tend to persist despite disconfirming positive information, a phenomenon linked to cognitive immunization, a phenomenon in which negative expectations persist despite contradictory positive experiences, and reduced belief updating [20, 21]. Anxiety may further amplify these processes by increasing attention to threat‐related information. These cognitive mechanisms are closely linked to expectation‐based models, which emphasize the role of predictive processes in shaping symptom perception and treatment outcomes [22]. In chemotherapy, anxiety is viewed as a key risk factor for side‐effect severity, particularly chemotherapy‐induced nausea (CIN), though findings remain heterogeneous [23]. In the context of treatment expectations and expectation‐related influences on chemotherapy‐related side effects, the constructs of hope and self‐efficacy warrant particular attention. Hope, as a multifaceted construct, refers to the confident expectation of achieving meaningful future goals [24], while cancer‐related self‐efficacy describes patients' belief in their ability to successfully manage the challenges associated with cancer and its treatment [25]. Theoretical models differentiate predictive expectations from constructs such as hope, which reflect desired future states rather than anticipated outcomes [26]. Nevertheless, hope and self‐efficacy may influence how patients perceive, interpret, and cope with treatment‐related side effects. In clinical oncology settings, hope has been shown to be dynamically shaped by patient–provider communication and to serve as an important coping mechanism, with patients often preferring positively framed information to sustain hope, even when facing advanced disease [27]. Both constructs have been associated with better psychological adjustment and coping during chemotherapy [28]. Hope has been linked to improved quality of life, social support, and spiritual well‐being in cancer patients across different disease stages [24, 29], while self‐efficacy correlates with improved symptom management and quality of life in oncology settings [30, 31].

Although previous research has demonstrated associations between treatment expectations, emotional distress, and chemotherapy‐related side effects, several important gaps remain. Existing research has focused predominantly on anxiety, nausea, and nocebo‐related mechanisms, while potentially protective psychological factors such as hope and self‐efficacy have received considerably less attention. In addition, findings regarding the relationship between side‐effect expectations and subsequent side‐effect severity remain inconsistent across symptom domains and study designs. Moreover, longitudinal research integrating psychological, social, and experiential factors within a broader symptom science perspective remains limited.

Drawing on the Theory of Unpleasant Symptoms (TOUS), the present study investigated the role of psychological factors (i.e., anxiety, depressive symptoms, hope, and self‐efficacy), prior treatment experiences, and social influences in shaping chemotherapy‐related side‐effect expectations and symptom severity over time. Anxiety and depressive symptoms were conceptualized as psychological influences on patients' expectations and symptom experiences. Chemotherapy‐induced nausea (CIN), chemotherapy‐induced peripheral neuropathy (CIPN), and chemotherapy‐related fatigue (CRF) served as the primary symptom outcomes. These symptom outcomes were selected because they represent highly prevalent and clinically burdensome chemotherapy‐related side effects that differ in their presumed relationship to psychological and expectation‐related processes [4]. CIN has frequently been linked to anticipatory and expectation‐related mechanisms [23], whereas CRF and CIPN have increasingly been associated with emotional distress, coping, and self‐management processes [24, 32].

Accordingly, the study addresses the following research questions:

1. To what extent are expectations of chemotherapy side effects associated with prior personal treatment experiences, secondary treatment experiences, and psychological factors (anxiety, depressive symptoms, hope, and self‐efficacy)?
2. Do side‐effect expectations and psychological factors predict the severity of chemotherapy‐related symptoms, specifically CIN, CIPN, and CRF?
3. What is the relative contribution of psychological factors, particularly hope and self‐efficacy, compared to established predictors such as prior experience and anxiety, in explaining side‐effect expectations and chemotherapy‐related symptom severity?

## 2 Methods

### 2.1 Study Design

The study employed a prospective longitudinal observational design with two measurement points. The study was conducted in accordance with the Declaration of Helsinki and received ethical approval from the Ethics Committee of the University of Heidelberg (reference number S‐124/2022). The study was registered in the German Clinical Trials Register (DRKS00028791). All patients provided written informed consent. The study was designed and reported in accordance with the STROBE (Strengthening the Reporting of Observational Studies in Epidemiology) guidelines [33]. A completed STROBE checklist is provided in the Supporting Information S1.

### 2.2 Patients and Study Procedure

The study sample consisted of patients with cancer beginning outpatient chemotherapy at the National Center for Tumor Diseases (NCT) in Heidelberg, Germany. Eligible patients were adults (≥ 18 years) with a current cancer diagnosis receiving outpatient treatment at the NCT, who were able to provide informed consent, had sufficient German language proficiency, and agreed to participate. Exclusion criteria included cognitive impairments affecting consent or questionnaire completion, severe psychiatric comorbidities (e.g., suicidality, dissociative disorders, complex trauma), or acute psychological distress with risk of decompensation. Patients were approached in person by study staff during treatment and informed about the study. Verbal and written information was provided, and written informed consent was obtained before any data collection. If participation was not possible or declined, the reason was documented. Consenting patients completed questionnaires at two time points: at the beginning of the second treatment cycle (T1) and 8–12 weeks later during the fourth cycle (T2), with treatment cycles defined as a phase of medication administration followed by a recovery phase. T1 was intentionally scheduled during the second cycle rather than the first to reduce patient burden during the initial treatment phase, which is typically characterized by a high number of clinical appointments (e.g., diagnostic procedures and treatment‐related information sessions) as well as elevated distress, uncertainty, and anxiety. This timing was also chosen to support study participation and data quality by avoiding additional burden at a particularly demanding time point.

### 2.3 Measures

At T1, sociodemographic data (age, sex, marital status) and secondary treatment experiences were assessed using a study‐specific questionnaire developed for this study. Secondary treatment experiences were assessed using three dichotomous (yes/no) items asking whether participants (1) had a close relative with cancer, (2) had been exposed to emotionally salient reports about cancer treatment from their social environment (e.g., fellow patients, family, friends, social media), and (3) had encountered such information through media sources such (e.g., internet, television, podcast or social media). Treatment‐related data (tumor site, disease stage, treatment phase, interval) were extracted from hospital records.

The *Generic Rating Scale for Previous Treatment Experiences, Treatment Expectations, and Treatment Effects* (G‐EEE [34], T1) provided a structured framework for assessing previous experiences, expectations, and effects. Only the items assessing prior chemotherapy side effects (“How many complaints/side effects have you experienced from chemotherapy in the past?”) and expected side effects (“How many complaints/side effects do you expect from chemotherapy?”) were used, referring specifically to the first treatment cycle. Responses ranged from 0 (= no complaints) to 10 (= greatest complaints imaginable). In the present study, these ratings were interpreted as indicators of patients' subjective symptom experiences related to chemotherapy side effects. For the present analyses, each item was treated as an individual continuous variable and entered separately into all statistical models.

The *Generic Assessment of Side Effects Scale* (GASE [35], T1 and T2) combined a 36‐item checklist with an open‐ended section for additional symptoms. Items reflected common pharmacological side effects (e.g., hair loss, nausea, fatigue). Peripheral neuropathy was added as a common chemotherapy side effect. Symptom intensity was rated on a four‐point Likert scale (0 = not present to 3 = severe), referencing the past 7 days.

The *Herth Hope Index*, German version (HHI‐D [36], T1), was used to assess multiple dimensions of hope in oncology settings. It consisted of 12 items rated on a four‐point Likert scale (0 = not at all true to 3 = very often true), yielding a total score from 0 to 36.

The *Cancer Behavior Inventory*, German version (CBI‐B‐D [37], T1), assessed coping‐related self‐efficacy beliefs. It comprised 14 items forming four subscales: (I) independence, (II) participation, (III) coping/stress management, and (IV) affect management. Patients rated their confidence in performing disease‐related coping behaviors on a nine‐point Likert scale (1 = not at all confident to 9 = completely confident), resulting in a total score from 14 to 126.

At T1, psychological distress was assessed using two ultra‐brief screening instruments: the *Patient Health Questionnaire‐2* (PHQ‐2 [38]) for depressive symptoms and the *Generalized Anxiety Disorder Scale‐2* (GAD‐2 [39]) for anxiety symptoms. The PHQ‐2 captured depressed mood and anhedonia; the GAD‐2 assessed nervousness/anxiety and difficulty controlling worry. Both used a four‐point Likert scale (0 = not at all to 3 = nearly every day) with total scores from 0 to 6. A cut‐off score ≥ 3 on either scale indicated clinically relevant symptom levels warranting further evaluation. In accordance with the conceptual framework of this study, PHQ‐2 and GAD‐2 scores were included as psychological predictor variables in the regression analyses.

Internal consistency of the multi‐item instruments was assessed using Cronbach's alpha. For the original validation studies, reported coefficients were α = 0.82 for the HHI‐D [36], α = 0.87 for the CBI‐B‐D [37], α = 0.75 for the PHQ‐2 [40], and α = 0.82 for the GAD‐2 [40]. In the present sample, Cronbach's alpha values were α = 0.80 for the HHI‐D, α = 0.95 for the CBI‐B‐D, α = 0.67 for the PHQ‐2, and α = 0.74 for the GAD‐2. Internal consistency was not calculated for the GASE, as the scale assesses a range of heterogeneous symptoms rather than a unidimensional construct. Similarly, no internal consistency coefficients were computed for the G‐EEE, as these measures are based on single‐item ratings. Secondary treatment experiences were treated as descriptive exposure characteristics rather than a latent construct; therefore, no formal psychometric validation or internal consistency assessment was performed.

### 2.4 Statistical Analyses

An a priori power analysis using G*Power (version 3.1.9.2 for Windows) indicated that a minimum of 109 patients was required to examine predictors of side‐effect expectations (eight predictors) and at least 98 to analyze predictors of specific side effects (six predictors), assuming a medium effect size (𝑓^2^ = 0.15), a target power of 0.80, and a significance level of 0.05. Based on the actual sample sizes included in the regression analyses, post hoc power calculations indicated an achieved power of approximately 0.75 for the model predicting side‐effect expectations (*n* = 98, 8 predictors) and approximately 0.64 for the models predicting specific side effects (*n* = 73, 6 predictors), assuming a medium effect size (f^2^ = 0.15) and α = 0.05.

Statistical analyses were performed in SPSS (version 28.0). Descriptive analyses were carried out for data collected at T1. To identify systematic differences between patients who dropped out after T1 and those who completed both assessments, chi‐square tests, Fisher's exact tests, and independent *t*‐tests were applied. Pearson's correlation coefficients were computed to examine associations between variables and assess potential multicollinearity. Multiple linear regression analyses were then used to investigate the effects of experienced side effects, secondary treatment experiences, hope, self‐efficacy, depression, and anxiety on side‐effect expectations. All predictors were entered simultaneously into the model. In addition, three separate hierarchical multiple regression analyses were conducted to examine the impact of side‐effect expectations and psychological variables assessed at T1 on nausea, peripheral neuropathy, and fatigue at T2. Because some patients had previously undergone chemotherapy, experienced side effects were entered in the first block of the model as control variables, followed by expected side effects and psychological variables in the second block. Model fit was evaluated by *R* ^2^ change statistics and the significance of incremental variance explained by each block. Standardized regression coefficients (*β*) were reported to facilitate interpretation of each predictor's relative contribution. Finally, no formal sensitivity analyses were conducted, as the limited sample size restricted the feasibility of subgroup or alternative model testing.

## 3 Results

### 3.1 Sample Characteristics and Study Completion

Recruitment was conducted between April and August 2023, and data collection was completed in October 2023. Prior to recruitment, all patients had been assessed for eligibility according to the predefined inclusion criteria. A total of 136 patients were approached, of whom 101 consented to participate. Non‐participation was mainly due to lack of interest, health‐related limitations, concerns about confidentiality, or negative prior experiences with research. Of those who consented, 98 patients completed the initial questionnaire (T1) in full; three cases were excluded due to missing values. Among these, 73 patients completed both the T1 and T2 questionnaires. Reported reasons for discontinuation after T1 included worsening health status, hospitalization, and limited time resources. Table 1 presents the sociodemographic, clinical, and psychological characteristics of the total sample, as well as separate data for completers and dropouts. Completers differed significantly from dropouts regarding scores on HHI‐D and CBI‐B‐D. A detailed overview of patient flow is provided in the Supporting Information S2.

**TABLE 1:** Sociodemographic, clinical and psychological characteristics of the total sample, completers and dropouts.

	Total sample (*n* = 98)	Complete data for T1 and T2 (*n* = 73)	Dropouts from T1 to T2 (*n* = 25)	Test‐statistic	*p*
Age					
Mean (SD)	51.21 (12.55)	53.54 (13.27)	46.63 (9.64)	T (98) = −2.62	0.053
Range	20–86	20–86	34–69		
Gender *n* (%)				χ2 (1) = 0.19	0.786
Male	15 (15.31)	12 (16.44)	3 (12.00)		
Female	83 (84.69)	61 (83.56)	22 (88.00)		
Marital status *n* (%)				*χ*2 (4) = 4.56	0.471
Single	11 (11.22)	9 (12.33)	2 (8.00)		
Divorced	8 (8.16)	5 (6.85)	3 (12.00)		
Widowed	6 (6.12)	6 (8.22)	0 (0.00)		
Cohabiting	14 (14.29)	11 (15.07)	3 (12.00)		
Married	59 (60.20)	42 (57.53)	17 (68.00)		
Tumor location *n* (%)				*χ*2 (4) = 5.76	0.343
Head/neck	4 (4.08)	3 (4.11)	1 (4.00)		
Gastrointestinal	16 (16.33)	11 (15.07)	5 (20.00)		
Urogenital	15 (15.31)	10 (13.70)	5 (20.00)		
Liver/pancreas	7 (7.14)	4 (5.48)	2 (8.00)		
Breast	56 (57.14)	44 (60.27)	12 (48.00)		
Lines of therapy *n* (%)				*χ*2 (1) = 0.02	0.994
First‐line‐therapy	84 (85.71)	62 (84.93)	22 (88.00)		
Second/third‐line‐therapy	14 (14.29)	11 (15.07)	3 (12.00)		
Cancer progression *n* (%)				*χ*2 (1) = 0.82	0.459
Non‐metastatic cancer	69 (70.41)	50 (68.49)	19 (76.00)		
Metastatic cancer	29 (29.59)	23 (31.51)	6 (24.00)		
Secondary treatment experience *n* (%)					
Close relative with cancer	48 (48.98)	38 (52.05)	10 (40.00)	*χ* ^2^ (1) = 0.52	0.523
Reports from social environment	25 (25.51)	19 (26.01)	6 (24.00)	*χ* ^2^ (1) = 0.01	0.999
Reports from media	34 (34.69)	21 (28.77)	12 (48.00)	*χ* ^2^ (1) = 3.11	0.113
CIN severity at T1 (GASE), Mean (SD)	1.06 (1.08)	0.95 (1.07)	1.42 (1.06)	T (98) = −1.87	0.064
CIPN severity at T1 (GASE), Mean (SD)	0.72 (0.97)	0.77 (0.97)	0.58 (0.97)	T (98) = 0.82	0.416
CRF severity at T1 (GASE), Mean (SD)	1.39 (0.89)	1.38 (0.92)	1.42 (0.83)	T (98) = −1.82	0.856
Experienced side effects (G‐EEE) Mean (SD)	5.37 (2.12)	5.38 (2.10)	5.37 (2.24)	T (98) = −0.01	0.825
Expected side effects (G‐EEE) Mean (SD)	2.97 (11.81)	1.98 (14.24)	5.00 (2.383)	T (98) = 1.18	0.190
Hope (HHI‐D) Mean (SD)	25.24 (8.76)	27.03 (3.78)	21.53 (13.75)	T (97) = −3.04	0.034
Self‐efficacy (CBI‐B‐D) Mean (SD)	91.81 (73.75)	101.57 (16.55)	71.68 (125.79)	T (96) = −1.91	0.018
Depression (PHQ‐2) Mean (SD)	1.43 (1.23)	1.21 (1.08)	1.90 (1.39)	T (98) = 2.69	0.541
Anxiety (GAD‐2) Mean (SD)	1.37 (1.25)	1.22 (1.06)	1.67 (1.34)	T (98) = 1.83	0.253

*Note:* SD = standard deviation; numbers in parentheses after test statistics indicate degrees of freedom; *p*‐values from the chi‐square test, Fisher's exact test, and independent *t*‐test.

Abbreviations: GASE (Generic Assessment of Side Effects Scale); G‐EEE (Generic Rating Scale for Previous Treatment Experiences, Treatment Expectations, and Treatment Effects); HHI‐D (Herth Hope Index, German version); CBI‐B‐D (Cancer Behavior Inventory, German version); PHQ‐2 (Patient Health Questionnaire‐2); GAD‐2 (Generalized Anxiety Disorder‐2).

### 3.2 Preliminary Correlations and Multicollinearity Diagnostics

Pearson correlation analyses were conducted to explore associations among the study variables and to assess potential multicollinearity (The bivariate correlations among the predictors are presented in the Supporting Information S3). A significant positive correlation was found between side‐effect expectations and secondary treatment experiences reported by the social environment (*r* = 0.26, *p* = 0.027), as well as with prior personal chemotherapy experience (*r* = 0.44, *p* < 0.001). Side‐effect expectations were also positively associated with depressive (PHQ‐2; *r* = 0.31, *p* = 0.009) and anxiety symptoms (GAD‐2; *r* = 0.27, *p* = 0.022), and negatively associated with hope (*r* = −0.26, *p* = 0.026). Furthermore, CIN severity correlated positively with both experienced (*r* = 0.21, *p* = 0.039) and expected side effects (*r* = 0.29, *p* = 0.007). CIPN severity was negatively associated with self‐efficacy (*r* = −0.22, *p* = 0.047) and positively associated with depressive symptoms (PHQ‐2; *r* = 0.37, *p* = 0.001). CRF severity showed a significant negative correlation with hope (*r* = −0.26, *p* = 0.027) and a positive correlation with depressive symptoms (PHQ‐2; *r* = 0.38, *p* = 0.001). Although correlations among psychological variables were observed, multicollinearity diagnostics were unremarkable: all tolerance values exceeded 0.1, and no variance inflation factor (VIF) values exceeded 10.

### 3.3 Predictors of Side‐Effect Expectations

A multiple regression analysis was conducted to examine associations between secondary treatment experience, experienced side effects, psychological variables, and side‐effect expectations at T1. The model was statistically significant (*F*(8, 89) = 3.87, *p* = 0.001), accounting for 42% of the variance in side‐effect expectations (*R* ^*2*^ = 0.42), indicating a large effect size (*f* ^*2*^ = 0.72). Experienced side effects of chemotherapy emerged as the strongest predictor (*β* = 0.47, *p* < 0.001). Among the psychological variables, significant effects were found for hope (*β* = −0.44, *p* < 0.001) and anxiety (*β* = 0.32, *p* < =0.018). Reports from the social environment were the only secondary treatment experience that were significantly associated with side‐effect expectations in the regression mode (*β* = 0.28, *p* < 0.024). Detailed results are presented in Table 2.

**TABLE 2:** Results of multiple regression analysis predicting side‐effect expectations at T1 from secondary treatment experience, experienced side effects, and psychological variables (*n* = 98).

Predictor	Bivariate correlation	Multiple regression analysis								
	*r* (*p*)	B	SE (B)	*β*	t (*p*)	95% CI for B		R	*R* ^2^	*F* (*p*)
						LB	UB			
Cancer in a relative	0.03 (0.782)	0.10	0.48	0.02	0.21 (0.838)	−1.1	0.86			
Social reports	0.26 (0.027)	1.47	0.64	0.28	2.32 (0.024)	0.57	1.93			
Media	0.00 (0.994)	−0.18	0.50	−0.04	−0.36 (0.722)	−1.2	0.96			
Experienced side effects (G‐EEE)	0.44 (0.000)	0.37	0.10	0.47	3.76 (0.000)	0.14	0.48			
Hope (HHI‐D)	−0.26 (0.026)	−0.24	0.06	−0.44	−3.80 (0.000)	−0.63	−0.11			
Self‐efficacy (CBI‐B‐D)	−0.20 (0.094)	0.29	0.31	−0.11	−0.74 (0.455)	−1.12	0.98			
Depression (PHQ‐2)	0.31 (0.009)	0.37	0.32	0.17	1.17 (0.248)	−0.18	0.51			
Anxiety (GAD‐2)	0.27 (0.022)	0.05	0.02	0.32	2.45 (0.018)	0.01	0.68			
								0.647	0.419	3.87 (0.001)

*Note: r* = Bravais‐Pearson bivariate correlation; *B* = unstandardized regression weight; *SE (B)* = standard error of B; *β* = standardized regression weight; *t* = *t* statistic testing whether B differs significantly from zero; LB = lower bound of the 95% confidence interval; UB = upper bound of the 95% confidence interval; *R* = multiple correlation coefficient; *R* ^*2*^ = proportion of variance explained by the model; *F* = *F* statistic from the analysis of variance test for the regression model.

Abbreviations: CBI‐B‐D, Cancer Behavior Inventory, German version; GAD‐2, Generalized Anxiety Disorder‐2 G‐EEE, Generic Rating Scale for Previous Treatment Experiences; HHI‐D, Herth Hope Index, German version; PHQ‐2, Patient Health Questionnaire‐2; Treatment Expectations, and Treatment Effects.

### 3.4 Predictors of Chemotherapy‐Related Symptom Severity (CIN, CIPN, CRF)

Three separate hierarchical multiple regression analyses were conducted to examine the influence of side‐effect expectations at T1 and psychological variables (hope, self‐efficacy, depression, and anxiety) on chemotherapy‐related symptom severity of CIN, CIPN and CRF, assessed at T2 using the GASE. The severity of previously experienced side effects was entered in Step 1 as a control variable to account for potential confounding effects. The first hierarchical multiple regression analysis examined predictors of CIN severity. In Step 1, the control variable (experienced side effects) did not significantly predict the outcome variable (*R* ^*2*^ = 0.05, *F*(1, 71) = 3.19, *p* = 0.078.) In Step 2, side‐effect expectations, hope, self‐efficacy, anxiety, and depressive symptoms were entered as predictors, resulting in a significant model, *R* ^*2*^ = 0.214, *ΔR* ^*2*^ = 0.17, *F*(5, 66) = 2.15, *p* = 0.044. The effect size for the final model was *f* ^*2*^ = 0.273, indicating a medium to large effect. However, none of the individual predictors in Step 2 reached statistical significance. For detailed results, see Table 3a.

**TABLE 3a:** Results of bivariate correlation and hierarchical multiple regression analysis predicting chemotherapy‐induced nausea (CIN) severity at T2 (*n* = 73).

Predictor	Bivariate correlation	Hierarchical multiple regression analysis									
	*r* (*p*)	B	SE (B)	*β*	t (*p*)	95% CI for B		R	*R* ^2^	Δ*R* ^2^	*F* (*p*)
						LB	UB				
Step 1:								0.21	0.05	0.05	3.19 (0.078)
Experienced side effects (G‐EEE)	0.21 (0.039)	0.073	0.04	0.21	1.78 (0.078)	−0.01	0.16				
Step 2:								0.46	0.21	0.17	2.15 (0.044)
Experienced side effects (G‐EEE)	0.21 (0.039)	0.04	0.05	0.10	0.77 (0.444)	−0.06	0.13				
Expected side effects (G‐EEE)	0.29 (0.007)	0.05	0.06	0.12	0.87 (0.385)	−0.05	0.19				
Hope (HHI‐D)	−0.13 (0.146)	0.02	0.04	0.10	0.57 (0.567)	.‐0.05	0.15				
Self‐efficacy (CBI‐B‐D)	−0.17 (0.081)	−0.01	0.01	−0.13	−0.81 (0.421)	−0.03	0.01				
Depression (PHQ‐2)	10 (0.383)	0.22	0.13	0.24	1.72 (0.091)	−0.05	0.45				
Anxiety (GAD‐2)	0.11 (0.372)	0.15	0.12	0.16	1.15 (0.256)	−0.07	0.47				

*Note: r* = Bravais‐Pearson bivariate correlation; *B* = unstandardized regression weight; SE (B) = standard error of B; *β* = standardized regression weight; *t* = *t* statistic testing whether B differs significantly from zero; LB/UB = lower/upper bounds of the 95% confidence interval; *R* = multiple correlation coefficient; *R* ^*2*^ = proportion of variance explained by the model; *ΔR* ^*2*^ = change in explained variance; *F* = *F* statistic from the analysis of variance test for the regression model.

Abbreviations: G‐EEE = Generic Rating Scale for Previous Treatment Experiences, Treatment Expectations, and Treatment Effects; HHI‐D = Herth Hope Index, German version; CBI‐B‐D = Cancer Behavior Inventory, German version; PHQ‐2 = Patient Health Questionnaire‐2; GAD‐2 = Generalized Anxiety Disorder‐2.

A second hierarchical multiple regression analysis was performed to investigate predictors of CIPN severity. In Step 1, the control variable (experienced side effects) did not account for a significant proportion of the variance (*R* ^*2*^ = 0.02, *F* (1, 71) = 1.48, *p* = 0.229.) In Step 2, the main predictors—side‐effect expectations, hope, self‐efficacy, anxiety, and depressive symptoms—were added, resulting in a significant increase in explained variance (*∆R* ^*2*^ = 0.18, *F* (5, 66) = 2.25, *p* = 0.042). The full model explained 20% of the variance in CIPN severity (*R* ^*2*^ = 0.20), with an effect size of *f* ^*2*^ = 0.253. Among the predictors, self‐efficacy (*β* = −0.31, *p* = 0.041) and depressive symptoms (β = 0.38, *p* = 0.007) emerged as significant contributors (see Table 3b).

**TABLE 3b:** Results of bivariate correlation and hierarchical multiple regression analysis predicting chemotherapy‐induced peripheral neuropathy (CIPN) severity at T2 (*n* = 73).

Predictor	Bivariate correlation	Hierarchical multiple regression analysis									
	*r* (*p*)	B	SE (B)	*β*	t (*p*)	95% CI for B		R	*R* ^2^	Δ*R* ^2^	*F* (*p*)
						LB	UB				
Step 1:								0.17	0.02	0.02	1.48 (0.229)
Experienced side effects (G‐EEE)	0.15 (0.114)	0.06	0.05	0.15	1.22 (0.229)	−0.04	0.15				
Step 2:								0.45	0.20	0.18	2.25 (0.042)
Experienced side effects (G‐EEE)	0.15 (0.114)	0.02	0.05	0.06	0.47 (0.637)	−0.05	14				
Expected side effects (G‐EEE)	0.09 (0.240)	0.02	0.06	0.04	0.30 (0.764)	−0.15	0.11				
Hope (HHI‐D)	−0.06 (0.303)	0.07	0.04	0.28	1.65 (0.104)	.‐0.03	0.13				
Self‐efficacy (CBI‐B‐D)	−0.22 (0.047)	−0.02	0.01	−0.31	−2.03 (0.041)	−0.04	−0.01				
Depression (PHQ‐2)	0.37 (0.001)	0.36	0.13	0.38	2.80 (0.007)	0.16	0.68				
Anxiety (GAD‐2)	0.07 (0.296)	−0.05	0.14	−0.05	−0.39 (0.669)	−0.41	0.12				

*Note: r* = Bravais‐Pearson bivariate correlation; *B* = unstandardized regression weight; *SE (B)* = standard error of B; *β* = standardized regression weight; *t* = *t* statistic testing whether B differs significantly from zero; LB/UB = lower/upper bounds of the 95% confidence interval; *R* = multiple correlation coefficient; *R* ^*2*^ = proportion of variance explained by the model; *ΔR* ^*2*^ = change in explained variance; *F* = *F* statistic from the analysis of variance test for the regression model.

Abbreviations: G‐EEE = Generic Rating Scale for Previous Treatment Experiences, Treatment Expectations, and Treatment Effects; HHI‐D = Herth Hope Index, German version; CBI‐B‐D = Cancer Behavior Inventory, German version; PHQ‐2 = Patient Health Questionnaire‐2; GAD‐2 = Generalized Anxiety Disorder‐2.

To investigate predictors of the severity of chemotherapy‐related fatigue (CRF), a third hierarchical multiple regression analysis was conducted. In Step 1, the control variable (experienced side effects) did not significantly explain variance in the outcome (*R* ^*2*^ = 0.04, *F* (1, 71) = 2.47, *p* = 0.106). When the main predictors—side‐effect expectations, hope, self‐efficacy, anxiety, and depressive symptoms—were entered in Step 2, the model showed a significant improvement in explanatory power (*∆R* ^*2*^ = 0.20, *F* (5, 66) = 2.72, *p* = 0.016). The final model accounted for 24% of the variance in CRF severity (*R* ^*2*^ = 0.24), with an effect size of *f* ^*2*^ = 0.312. Among the predictors, hope (*β* = −0.41, *p* = 0.036) and depressive symptoms (*β* = 0.39, *p* = 0.005) were identified as significant contributors (Table 3C).

**TABLE 3C:** Results of bivariate correlation and hierarchical multiple regression analysis predicting chemotherapy‐related fatigue (CRF) severity at T2 (*n* = 73).

Predictor	Bivariate correlation	Hierarchical multiple regression analysis									
	*r* (*p*)	B	SE (B)	*β*	t (*p*)	95% CI for B		R	*R* ^2^	Δ*R* ^2^	*F* (*p*)
						LB	UB				
Step 1:								0.19	0.04	0.04	2.68 (0.106)
Experienced side effects (G‐EEE)	0.19 (0.053)	0.06	0.04	0.19	1.64 (0.106)	−0.01	0.14				
Step 2:								0.49	0.24	0.20	2.72 (0.016)
Experienced side effects (G‐EEE)	0.19 (0.053)	0.07	0.04	0.22	1.59 (0.095)	−0.10	0.15				
Expected side effects (G‐EEE)	−0.01 (0.485)	−0.08	0.06	−0.21	−1.45 (0.152)	−0.19	0.03				
Hope (HHI‐D)	−0.26 (0.027)	0.08	0.04	−0.41	2.10 (0.036)	0.01	0.13				
Self‐efficacy (CBI‐B‐D)	−0.16 (0.097)	−0.01	0.01	−0.21	−1.36 (0.179)	−0.20	0.02				
Depression (PHQ‐2)	0.38 (0.001)	0.32	0.11	0.39	2.89 (0.005)	0.11	0.54				
Anxiety (GAD‐2)	0.21 (0.945)	0.065	0.11	0.07	0.54 (0.585)	−0.16	0.28				

*Note: r* = Bravais‐Pearson bivariate correlation; *B* = unstandardized regression weight; *SE (B)* = standard error of B; *β* = standardized regression weight; *t* = *t* statistic testing whether B differs significantly from zero; LB/UB = lower/upper bounds of the 95% confidence interval; *R* = multiple correlation coefficient; *R* ^*2*^ = proportion of variance explained by the model; *ΔR* ^*2*^ = change in explained variance; *F* = *F* statistic from the analysis of variance test for the regression model.

Abbreviations: G‐EEE = Generic Rating Scale for Previous Treatment Experiences, Treatment Expectations, and Treatment Effects; HHI‐D = Herth Hope Index, German version; CBI‐B‐D = Cancer Behavior Inventory, German version; PHQ‐2 = Patient Health Questionnaire‐2; GAD‐2 = Generalized Anxiety Disorder‐2.

## 4 Discussion

This study examined the influence of side‐effect experiences, secondary treatment experiences, and psychological factors (hope, self‐efficacy, anxiety, depression) on expectations of chemotherapy‐related side effects and patients' symptom experiences of CIN, CIPN, and CRF. Our results show that experienced side effects were the strongest predictor of expectations, followed by the psychological factors hope and anxiety and the reports from the social environment. Regarding symptom severity, self‐efficacy and depressive symptoms predicted CIPN severity, whereas hope and depressive symptoms predicted CRF severity. In contrast to previous research, neither side‐effect expectations nor anxiety were linked to the severity of side effects. These findings support a symptom science perspective in which chemotherapy‐related symptom experiences emerge from the interplay of psychological, situational, and treatment‐related influences rather than from expectations alone.

Previous evidence indicates that adults receiving outpatient chemotherapy typically experience a multidimensional symptom burden, with fatigue consistently being among the most prevalent and burdensome symptoms, while gastrointestinal and psychological symptoms are also commonly reported [40, 41]. Consistent with these reports, the overall symptom burden observed in the present sample was comparable to that described in previous outpatient oncology populations, with CRF showing the highest mean severity, followed by CIN and CIPN. The comparatively low severity of CIPN may reflect the timing of assessment, as participants were evaluated at the beginning of the second chemotherapy cycle, before the cumulative effects of neurotoxic treatment would typically be expected [42]. This interpretation is further supported by longitudinal studies demonstrating that chemotherapy‐related symptoms follow different trajectories over the course of treatment, with fatigue and nausea tending to fluctuate across cycles, whereas CIPN accumulates with continued exposure to neurotoxic agents [43, 44].

### 4.1 Experience, Social Influence, and Expectation Effects

Consistent with previous research [13, 14, 45], our findings confirm that patients' expectations about chemotherapy side effects are strongly shaped by their earlier symptom experiences. In addition to personal experience, reports from the social environment—such as accounts from fellow patients, family members, or social media—also emerged as a relevant predictor of side‐effect expectations. These influences may operate through mechanisms such as expectation shaping, heightened symptom monitoring, and altered symptom interpretation [15]. Socially transmitted information can take many forms—including verbal reports, symbolic representations, and video modeling—and is increasingly mediated by social media and online forums [46].

We observed a correlation between nausea expectations and subsequent experience, but not with overall severity of side effects. This partly contrasts with a review by Smith et al. [47], which identified expectations as strong predictors of later symptom perception, particularly nausea, and linked them to treatment non‐adherence. At the same time, our results are consistent with research showing more differentiated associations [48]. In that work, nausea expectations were associated with prior experiences and anxiety but did not independently predict post‐chemotherapy nausea once other clinical factors such as age and baseline quality of life were considered. Methodological variability in instruments, response scales, and timing of assessment may also help explain divergent findings.

### 4.2 Hope, Self‐Efficacy, and Emotional Distress

In the present study, higher levels of hopeful thinking were associated with lower expectations of treatment‐related side effects. Hope also emerged as both a correlate and a predictor of fatigue, with lower hope linked to greater fatigue severity. Patients who dropped out of the study reported lower hope at T1. This pattern may be understood in light of theoretical models distinguishing predictive expectations—which are central to expectation‐related symptom processes—from constructs such as hope, which reflect desired future states rather than anticipated outcomes and may therefore influence symptom perception more indirectly [26]. Our findings align with previous research showing inverse associations between hope and symptoms such as fatigue, dyspnea, and pain [48]. Evidence further suggests a bidirectional relationship: increases in hope—particularly the ability to generate and maintain flexible strategies for reaching goals despite obstacles—may reduce daily fatigue, whereas persistent fatigue can undermine hope, especially the sense of agency [49]. While often discussed alongside optimism, hope differs in that optimism reflects a general expectation of positive outcomes, whereas hope implies that such outcomes can be achieved through one's own actions [50]. The protective role of hope may also be understood in contrast to catastrophizing, a maladaptive cognitive style marked by exaggerated negative appraisals and helplessness. Hope and catastrophizing may represent opposing ends of a cognitive‐emotional continuum, with hope fostering active coping and catastrophizing reinforcing anxiety and withdrawal [51].

No significant effect of self‐efficacy on the expectation of side effects was found, but self‐efficacy correlated with and predicted CIPN. Lower self‐efficacy was also associated with study dropout. These findings correspond with prior research showing that individuals with neuropathic pain often report lower self‐efficacy and higher depression, both hindering effective self‐management [52]. In oncology, self‐efficacy is increasingly recognized as crucial for managing CIPN and is linked to relevant self‐care behaviors [53, 54]. This appears plausible given the invasive nature of chemotherapy, where side effects arise early and are clearly attributable to treatment. In such contexts, expectations may be less influential than patients' capacity to cope with symptoms. Self‐efficacy may therefore influence symptom outcomes primarily through coping‐related mechanisms, while playing a more indirect role in the formation of treatment expectations.

The findings also indicate distinct roles of anxiety and depression in shaping symptom expectations and experiences. These patterns may be interpreted within predictive processing frameworks, suggesting that depression is associated with persistent negative expectations due to biased belief updating and cognitive immunization, while anxiety amplifies threat‐related processing [20, 21]. Anxiety was associated with expectations of chemotherapy side effects but did not predict their actual occurrence. In contrast, depressive symptoms were unrelated to expectations but predicted both CIPN and CRF. These mixed patterns reflect the inconsistent literature, where associations between emotional distress and treatment‐related symptom experience vary considerably [47]. For anxiety, effects may depend on how the construct is defined, and higher anxiety might even prompt greater engagement with supportive care or prophylactic measures, potentially buffering against symptom development. The robust link between depression and CRF, however, is well established, with depressive symptoms consistently associated with higher risk and greater fatigue severity [55]. Clinical interpretation remains difficult due to conceptual and symptomatic overlap between fatigue and depression, which can lead to diagnostic uncertainty and misattribution of symptoms [56, 57].

### 4.3 Clinical Implications

Negative expectations may be associated with increased psychological vulnerability and heightened perception related to chemotherapy side effects and should therefore be addressed through sensitive communication and supportive care. Because prior treatment experiences and socially transmitted information strongly shape patients' expectations, it is important to explore these influences and respond with transparent, empathetic dialog. Patients are increasingly exposed to reports from peers, family members, and online communities; discussing such narratives, validating concerns, and providing balanced information about treatment‐related side effects may help reduce distress and excessive symptom anticipation. Exploring patients' prior treatment experiences and side‐effect expectations early in the treatment process may help clinicians better understand individual concerns and supportive care needs during chemotherapy.

Self‐efficacy and hope may play an important role in shaping symptom perception and adjustment. Psycho‐oncological programs may therefore benefit from integrating interventions that strengthen self‐efficacy and enhance hope as a motivational and cognitive process. Monitoring hope and self‐efficacy during chemotherapy may also assist in identifying patients at risk for low adherence or early dropout, allowing timely supportive intervention. Finally, screening for and treating depressive symptoms remains essential, as depression consistently predicted greater symptom burden in this study.

From a research perspective, the lack of consistent links between expectations and symptom severity underscores the need for more nuanced investigation. Larger and more diverse samples are needed to clarify how hope and self‐efficacy interact with clinical factors such as cancer type, stage, or prognosis. Future work should also disentangle different aspects of anxiety (state, trait, health‐related) to specify their role in symptom expectations and outcomes.

### 4.4 Study Limitations

Several limitations must be considered. First, the final sample was smaller than anticipated in the a priori power analysis. Post hoc power considerations suggest that the achieved statistical power was slightly below the conventional threshold, which may have limited the ability to detect smaller effects. Recruitment proved challenging in this clinically burdened population, and many eligible patients declined participation, often citing the questionnaire battery as too demanding. This may have introduced self‐selection bias and limited generalizability.

Second, T1 was assessed during the second chemotherapy cycle rather than at treatment initiation. This timing was selected to minimize additional demands during the initial phase of chemotherapy, which is typically characterized by a high density of clinical procedures and increased emotional strain. While this approach was intended to support participation and data quality, it may have limited the assessment of very early treatment‐related reactions. In particular, initial fluctuations in symptom burden or distress during the first cycle may not be fully reflected. The findings should therefore be interpreted as capturing patients' experiences after an initial phase of adjustment rather than their immediate responses to treatment onset. In addition, dropout analyses indicated that patients with lower self‐efficacy, lower hope, and younger age were more likely to discontinue participation, resulting in a final sample with a narrower range of hope, self‐efficacy, and age, which may have attenuated the observed associations.

Moreover, emotional distress was assessed with ultra‐brief screening tools (PHQ‐2, GAD‐2). Although validated in oncology, these instruments capture only core symptoms and do not represent the full spectrum of affective difficulties. Brevity was chosen to minimize burden and to maintain focus on self‐efficacy and hope, but necessarily limited content coverage. In this context, it should also be noted that internal consistency was acceptable to excellent for most instruments, whereas the PHQ‐2 showed somewhat lower internal consistency in the present sample (α = 0.67). This is consistent with previous research on ultra‐brief measures and reflects the limited number of items rather than insufficient measurement quality; however, findings related to depressive symptoms should be interpreted with some caution. In addition, only selected subscales of the GASE and G‐EEE were applied, which reduces comparability with studies using the full instruments. Furthermore, the study‐specific questionnaire assessing secondary treatment experiences was not formally validated and captured categorical (yes/no) exposure variables.

Although no statistically significant differences were observed across clinical characteristics such as tumor site, metastatic status, and treatment line, potential clinical heterogeneity cannot be ruled out. Differences in multimorbidity, comorbidity burden, treatment regimen characteristics, supportive medication, disease burden, and prognosis may have influenced both psychological variables and treatment‐related outcomes. These variables were not included in the regression models and may have influenced symptom occurrence and severity. Therefore, residual confounding cannot be excluded. Furthermore, information on symptom management interventions (e.g., supportive care measures or symptom‐specific treatments) was not systematically collected and could therefore not be considered in the analyses. Such interventions may have influenced symptom occurrence and severity and should be taken into account in future studies. Due to the limited sample size, these clinical variables were not included as covariates in the regression analyses, as this would have increased the risk of model overfitting and reduced statistical power. Consequently, the observed effects of psychological predictors should be interpreted with significant caution. Finally, the single‐centre design, relatively small sample size, and the inclusion of patients assessed at the same stage of chemotherapy restrict external validity; the results may not generalize to other treatment phases, cancer types, or clinical settings.

Despite these limitations, the study has several strengths that support the robustness of its findings. Its longitudinal design within a routine clinical setting enabled observation of symptom changes over time and allowed cautious interpretations regarding potential causal relationships. A further strength was that all participants were assessed at the same stage of chemotherapy, thereby reducing variability related to treatment timing. Moreover, the inclusion of psychological constructs such as hope and self‐efficacy—rarely examined in this context—provided an additional contribution to understanding patient‐related determinants of symptom perception.

## 5 Conclusions

This study found that expectations of chemotherapy side effects were shaped by prior chemotherapy experience, anxiety, lower hope, and negative treatment reports from others, highlighting the combined role of personal, social, and psychological factors. However, the relationship between side‐effect expectations and subsequent symptom severity was less consistent than anticipated. While side‐effect expectations were associated with nausea at the bivariate level, they did not independently predict symptom severity in the multivariable analyses. Among the psychological variables, lower hope predicted greater fatigue, depressive symptoms independently predicted both nausea and fatigue severity, and self‐efficacy was associated with lower neuropathy and continued study participation. These findings suggest that hope and self‐efficacy may serve complementary protective functions across different chemotherapy‐related symptoms, though confirmation in larger and more diverse samples is needed given the limited sample size, selective attrition, and use of ultra‐brief screening instruments. The findings further suggest that psychological resilience factors may play a greater role in patients' symptom experiences than treatment expectations alone. Clinically, the results support integrating expectation management, hope‐strengthening approaches, and depression screening into psycho‐oncological care, ideally beginning prior to the first chemotherapy cycle.

## Author Contributions

**Hans‐Christoph Friederich:** writing – review and editing, supervision. **Till Johannes Bugaj:** writing – review and editing, supervision, project administration. **Miriam Grapp:** conceptualization, investigation, writing – original draft, formal analysis, methodology. **Kristin Lichtenberg:** conceptualization, investigation, methodology, formal analysis, writing – review and editing. **Marcel Wilhelm:** writing – review and editing, supervision.

## Conflicts of Interest

The authors declare no conflicts of interest.

## Supporting information


**Supporting Information: S1** STROBE Statement—checklist of items that should be included in reports of observational studies.

**Supporting Information: S2** Patient Flow Chart.

**Supplementary Information: S3** Overview of the bivariate correlations among all predictors considered in the analyses.

## Data Availability

The data that support the findings of this study are available from the corresponding author upon reasonable request.
