# Supplementary figures and images for: Psychological Factors and Treatment Experiences Associated With Chemotherapy Side‐Effect Expectations and Symptom Severity: A Prospective Longitudinal Study
Source: Cancer Med. 2026 Jul 14;15(7):e72117. doi: 10.1002/cam4.72117 (PMC13369292; doi:10.1002/cam4.72117)

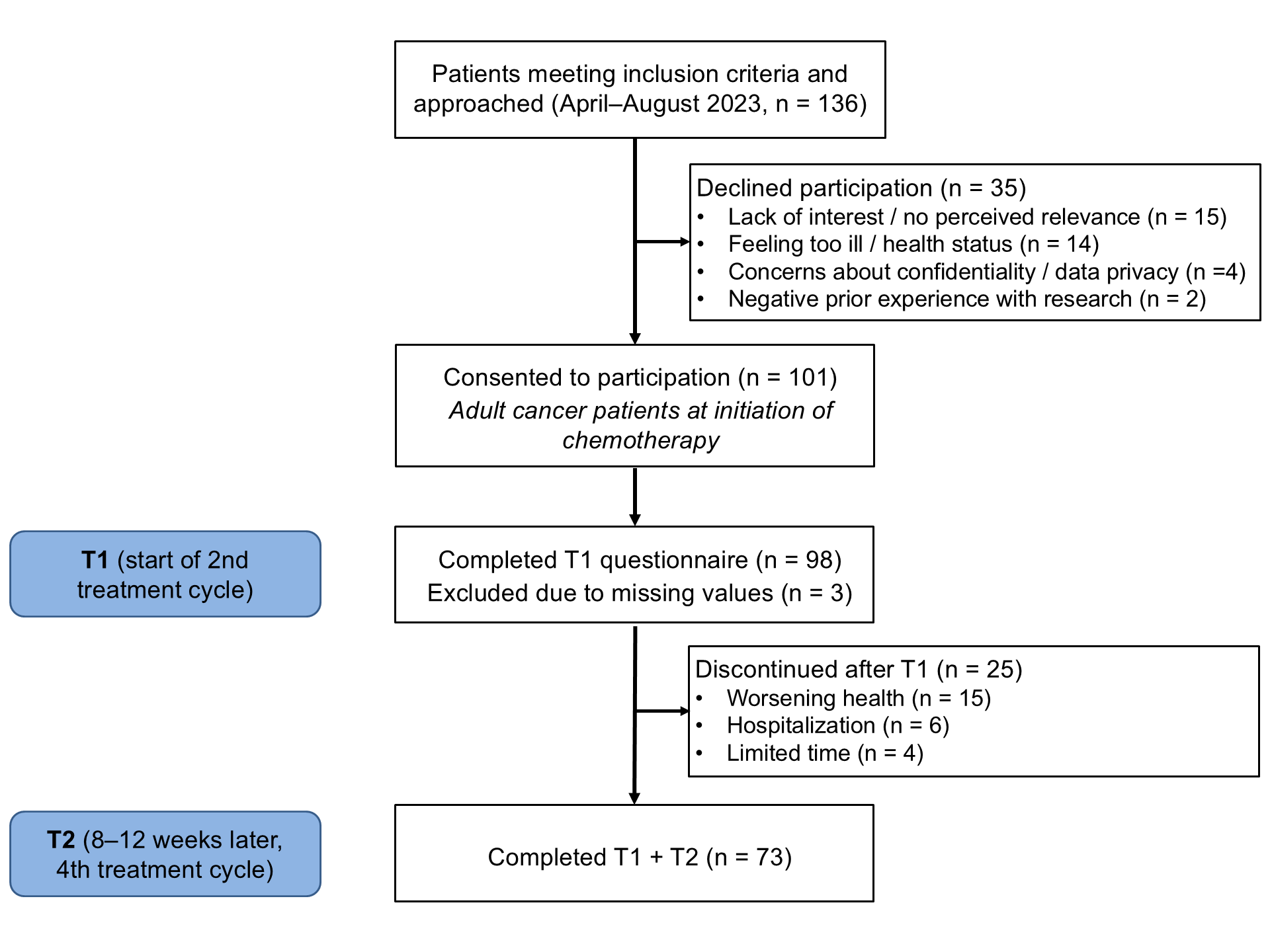

Supplement: Supplementary file 2 — Supporting Information: S2 Patient Flow Chart. [file CAM4-15-e72117-s002.tif]
