# Supplementary material for: Psychological Factors and Treatment Experiences Associated With Chemotherapy Side‐Effect Expectations and Symptom Severity: A Prospective Longitudinal Study
Source: Cancer Med. 2026 Jul 14;15(7):e72117. doi: 10.1002/cam4.72117 (PMC13369292; doi:10.1002/cam4.72117)
Supplement: Supplementary file 3 — Supplementary Information: S3 Overview of the bivariate correlations among all predictors considered in the analyses. [file CAM4-15-e72117-s003.docx]

**Supplementary Information 3. Overview of the bivariate correlations among all predictors considered in the analyses**

| **Predictor** | **1** | **2** | **3** | **4** | **5** | **6** | **7** | **8** | **9** |
| --- | --- | --- | --- | --- | --- | --- | --- | --- | --- |
| **1. Experienced side effects (G-EEE)** | – |  |  |  |  |  |  |  |  |
| **2. Side-effect expectations at T1 (G-EEE)** | .44** | – |  |  |  |  |  |  |  |
| **3. Knowledge from observing a close relative with cancer** | -.02 | .03 | – |  |  |  |  |  |  |
| **4. Knowledge from social environment reports** | -.12 | .26* | .06 | – |  |  |  |  |  |
| **5. Knowledge from media/other sources** | .11 | .00 | -.10 | .27* | – |  |  |  |  |
| **6. Self-efficacy (CBI-B-D)** | -.08 | -.20 | .10 | -.07 | -.00 | – |  |  |  |
| **7. Hope (HHI-D)** | -.08 | -.26* | .10 | .23 | .10 | .56** | – |  |  |
| **8. Depression (PHQ-2)** | .17 | .31** | .06 | -.05 | .19 | -.33** | -.32** | – |  |
| **9. Anxiety (GAD-2)** | .11 | .27* | -.03 | .08 | .09 | -.33** | -.27* | .41** | – |

Note. Values are Pearson correlation coefficients. *p* < .05 (*), *p* < .01 (**).
